# Feasible Cluster Model Method for Simulating the Redox Potentials of Laccase CueO and Its Variant

**DOI:** 10.3389/fbioe.2022.957694

**Published:** 2022-07-22

**Authors:** Qixuan Jiang, Ziheng Cui, Ren Wei, Kaili Nie, Haijun Xu, Luo Liu

**Affiliations:** ^1^ Beijing Bioprocess Key Laboratory, Beijing University of Chemical Technology, Beijing, China; ^2^ Junior Research Group Plastic Biodegradation at Institute of Biochemistry, University of Greifswald, Greifswald, Germany

**Keywords:** cluster model, redox potential, quantum mechanics, laccase, molecular simulation

## Abstract

Laccases are regarded as versatile green biocatalysts, and recent scientific research has focused on improving their redox potential for broader industrial and environmental applications. The density functional theory (DFT) quantum mechanics approach, sufficiently rigorous and efficient for the calculation of electronic structures, is conducted to better comprehend the connection between the redox potential and the atomic structural feature of laccases. According to the crystal structure of wild type laccase CueO and its variant, a truncated miniature cluster model method was established in this research. On the basic of thermodynamic cycle, the overall Gibbs free energy variations before and after the one-electron reduction were calculated. It turned out that the trends of redox potentials to increase after variant predicted by the theoretical calculations correlated well with those obtained by experiments, thereby validating the feasibility of this cluster model method for simulating the redox potentials of laccases.

## Introduction

Laccases (EC 1.10.3.2) are oxidoreductases with cupredoxin-like domains that can catalyze various substrates oxidized and simultaneously reduce oxygen to water (Madhavi and Lele, 2009; Janusz et al., 2020; Shiroya, 2021). Laccases contain at least four copper atoms comprising one type 1 copper (T1 Cu) serving as the electron entry position to the protein, and one type 2 copper (T2 Cu) and two type 3 coppers (T3 Cu) that compose a trinuclear cluster (TNC) (Sakurai and Kataoka, 2007; Solomon et al., 2008; Liu et al., 2014; Giacobelli, 2017). Given the apparent stability and eco-friendliness of laccases, scientific efforts have been diverted these days to exploit such enzymes in emerging fields such as enzymatic biofuel cells and the degradation of noxious contaminants (Bilal et al., 2019; Li et al., 2020; Moreno et al., 2020). The redox potentials of laccases generally range from 0.43 to 0.79 V versus the normal hydrogen electrode (NHE) (Mateljak et al., 2019). Through directed evolution of laccases, the redox potential value is expected to be improved to fulfill the requirement for many biotechnological and environmental applications.

The copper efflux oxidase (CueO) has conspicuously superior stability in high temperature or alkaline environment when compared to other bacterial laccases. Nevertheless, its relatively low redox potential is detrimental to catalytic activity from a thermodynamic perspective (Santhanam et al., 2011; Martins et al., 2015; Chauhan et al., 2017). Two beneficial substitutions D439T and L502K were found to improve the onset potential up to 0.42 and 0.44 V, respectively (Zhang et al., 2019). The crystal structure of CueO revealed that D439 and L502 form hydrogen bonds with coordinated residues of T1 Cu, H443 and C500, respectively. The redox potential of CueO is mostly governed by the T1 Cu site (Hong et al., 2011; Vázquez-Lima et al., 2012). Computational simulation of the redox potential variation influenced by single-site variants adjoining the T1 Cu site is one interesting avenue of investigation.

Selecting appropriate methods and developing a computational scheme for catalytic processes associated with redox-active metalloenzymes is a challenging task. With the rapid development of computing science, numerous multiscale modeling approaches for enzymes have been adopted, including molecular dynamics (MD), free energy perturbation (FEP), empirical valence bond (EVB), hybrid quantum mechanics/molecular mechanics (QM/MM), and the quantum mechanics (QM) cluster approach (Ahmadi et al., 2018; Sheng et al., 2020). Supposing that the selected QM region is reasonable, the same consequences will be given by QM cluster and QM/MM approaches essentially (Siegbahn, 2006; Liao and Thiel, 2012). The cluster approach is the simple and efficient method for elucidating mechanisms of redox-active metalloenzymes, dealing with the crucial active site region of the metalloenzymes in a QM manner, proved fruitful in reproducing high-precision energy calculations. Lately, Mina Ghiasi et al. (2021) carried out a DFT research on the activation mechanism of the human carbonic anhydrase VII cluster model and demonstrated that the activator molecule participates in proton transfer reactions, enhancing the formation of the active zinc hydroxide species.

In this work, we developed a T1 Cu site active center cluster model protocol for calculating the redox potentials of WT CueO and its variant L502K. The dynamics simulations were carried out, and according to the obtained equilibrium structures, the target cluster model structures were designed, whose oxidized and reduced states were geometrically optimized separately in the solvated environment at the B3LYP-D3(BJ)/6-311G* level. Their vapor phase and solvation Gibbs free energies were calculated at the M062X/def2TZVP and M06X/6-311G* level, respectively, to achieve the redox potentials. Several different DFT and solvation approaches were attempted during the simulation to achieve more accurate results.

## Methods

### Quantum Mechanics Cluster Model Approach

The QM cluster model approach, which uses finite models to investigate the active sites of metalloenzymes, is an important tool to elucidate enzymatic reaction mechanisms. Many aspects of the metalloenzyme mechanisms have been understood, employing a relatively small model (Blomberg et al., 2014).

Density functional theory (DFT), a currently used QM atomistic simulation method, can be utilized to describe the electronic structure of the enzyme cluster model system (Himo, 2017). The hybrid functional B3LYP has dominated in the geometric optimization applications, accounting for its simple form, low dependence on the integration lattice point, fast calculation speed, and moderate accuracy. Nonetheless, the B3LYP functional is not suitable for describing weak interactions caused by dispersion (Grimme et al., 2016). In this respect, the recent developed technique, adding an empirical dispersion correction, known as DFT-D, has been shown to dramatically improve the energies in the field of homogeneous catalysis (Witte et al., 2015). When it comes to energy calculations, the M06 functional appears to offer better performance in predicting the overall trends of relative energies than the B3LYP functional (Walker et al., 2013).

Since the cluster approach contains only an enzyme fraction, namely, the active site and its surroundings, when the structures are designed, environment influences need to be taken into account. This environment impact is usually simulated by two simple approximations: steric hindrance and static electricity. To prevent the overall structure of the model from unraveling during optimization, some atoms at the model boundary are fixed. The electrostatic effect of solvent contribution is approximated by the implicit solvation model. The implicit solvation model treats the solvent as a dielectric continuum, in which different solvation environment can be represented by regulating permittivity ***ε***. Furthermore, it should be noted that as the model size increases, the approximation improves. With these two approximations, a cluster model suitable for the T1 Cu site active center of WT CueO and its variant can be constructed.

### Theoretical Methods for Calculating Redox Potentials

**SCHEME 1:**
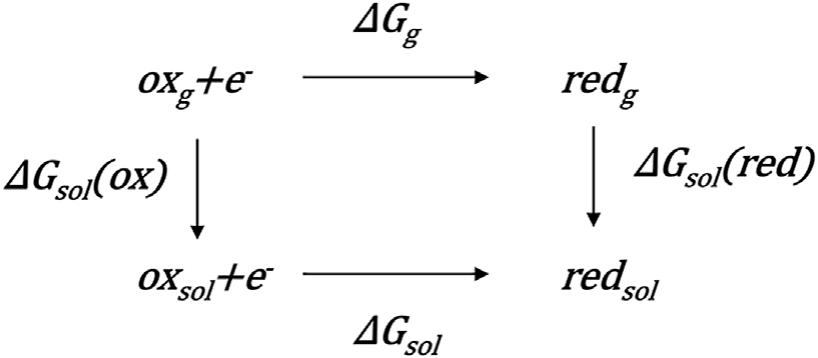
Thermodynamic cycle to calculate the one-electron reduction potentials in solution.

The theoretical estimate for the concerned potential of the redox reaction ( $\mathrm{ox}+e^{-}\to \mathrm{red}$ ) is

$$-\Delta E=-\frac{\Delta G}{\mathrm{nF,}}$$ (1)

where ΔE is the redox potential, ΔG is the variation of Gibbs free energy, n is the number of electrons, and F is the Faraday constant (Bruschi et al., 2016). The main problem associated with the calculation is that the reactions always take place in solution, whereas directly calculating the solvated Gibbs free energy is low in accuracy (Yan et al., 2016). It is well known that the accuracy of gas phase energy is higher than that of liquid phase energy in QM calculations; consequently, the thermodynamic cycle in Scheme 1 and the following formula are proposed to exactly calculate the ΔG value,

$$\mathrm{\Delta G}=\Delta G_{g}+\Delta \Delta G_{\mathrm{sol}}$$ (2)

where $\Delta G_{g}=G_{g}\left(\mathrm{red}\right)-G_{g}\left(\mathrm{ox}\right)$ is the gas phase Gibbs free energy difference and $\Delta \Delta G_{\mathrm{sol}}=\Delta G_{\mathrm{sol}}\left(\mathrm{red}\right)-\Delta G_{\mathrm{sol}}\left(\mathrm{ox}\right)$ is the differential free energy of solvation (Uudsemaa and Tamm, 2003). The Gibbs free energy for oxidized and reduced states in the gas phase are calculated respectively to hence the $\Delta G_{g}$ value. An implicit solvent model is used for both states yielding the differential solvation energy $\Delta \Delta G_{\mathrm{sol}}$ (Holland et al., 2006). The redox potential can eventually be obtained by Eqs 1 and 2.

### Molecular Dynamic Simulations

The crystal structure of CueO resolved at an atomic resolution of 1.1 Å (PDB code: 3OD3) was used as a starting point of MD simulations (Singh et al., 2011). The structure of the L502K variant was generated using the WT CueO structure as a template by YASARA software version 17.8.15 (Krieger and Vriend, 2014). Molecular dynamics (MD) simulation and analysis were performed with NAMD software version 2.14 (Phillips et al., 2020). Protonation statuses of residues were determined by the H++ online program and checked manually (Anandakrishnan et al., 2012). A cubic box with a protein-to-border distance of 10 Å was established and filled with TIP3P water molecules. The systems were minimized and equilibrated for 20 ns with the T1 Cu site kept fixed. Root mean square deviation (RMSD) was calculated by VMD package tools (Phillips et al., 2020).

### Quantum Chemical Calculations

The quantum chemical calculations for optimization and vibration analysis of the system were performed at the B3LYP-D3(BJ)/6-311G* level of theory combined with the SMD solvent model. The total charge of oxidized and reduced was 1 and 0, respectively, because the amino acid ligands are neutral, while the SG atom in C500 is coordinated with copper ions, leading to the HG atom originally attached to the SG atom deprotonated. The spin multiplicity of oxidized and reduced was 2 and 1, respectively. More precise electron energies in vacuum were calculated at the M062X/def2TZVP level dependent on the optimized structures. Simultaneously, the M062X/6-311G* level was selected for calculating the solvation free energy difference. All quantum mechanical calculations were carried out using the Gaussian 16 program package (Frisch et al., 2016).

## Results and Discussion

### Molecular Dynamic Simulations

**FIGURE 1:**
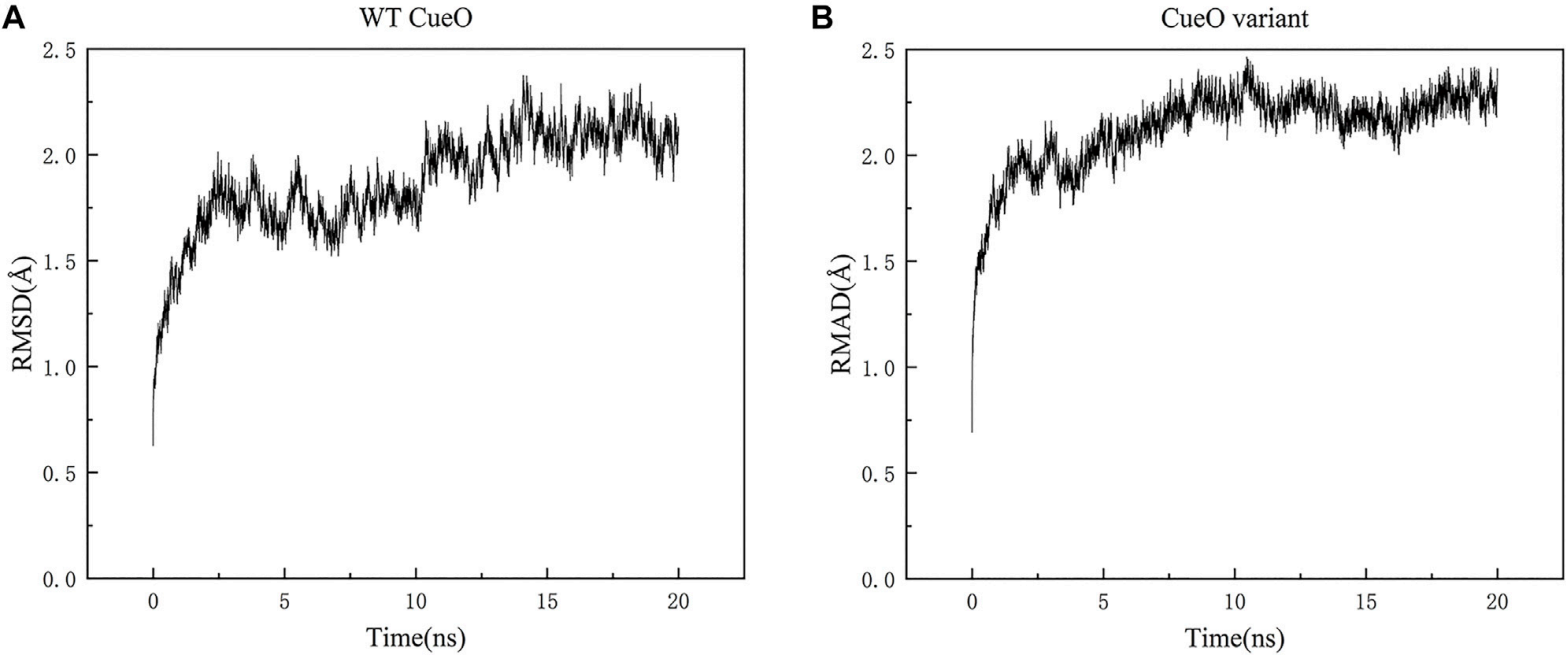
Backbone RMSD for **(A)** WT CueO and **(B)** the L502K variant during 20 ns course of the MD simulations.

Molecular dynamic simulations testing on both structures (WT CueO L502K variant) showed good results in Figure 1. The backbone RMSD of the equilibrated structures relative to the original structures range from 0.63 to 2.38 Å and 0.69 to 2.46 Å for WT CueO and the L502K variant, respectively.

### Energetics of the Catalytic Site

**FIGURE 2:**
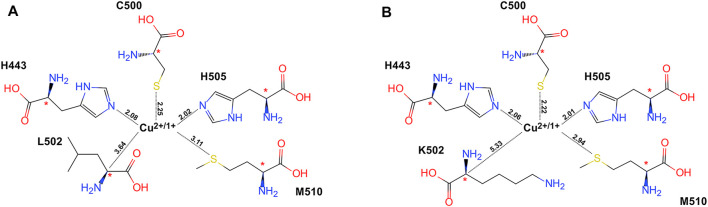
Schematic illustration of the cluster model system based on equilibrated crystallographic data collected by molecular dynamic simulation, with key atoms labeled. The stars indicate the atoms that were frozen during the optimization procedure. **(A)** WT CueO and **(B)** the L502K variant. The key distances between cupper atom and amino acids were drawn with dotted lines, and the values were labeled (the unit is Å).

The cluster models for QM research studies were constructed with the residues involved in the biocatalysis active site. The copper ion (Cu^2+^ or Cu^1+^) in the core and its ligands (H443, C500, and H505) and L502 for the WT or K502 for the variant, as well as the hydrophobic residues M510, were considered in the enzyme cluster model as shown in Figure 2. After a consolidating procedure in the cluster model, the α carbon atoms of all residues were fixed in their positions at molecular dynamic equilibrium dependent on the crystal structure during the geometry optimization for the sake of avoiding cluster model discretization inconsistent with actuality.

**FIGURE 3:**
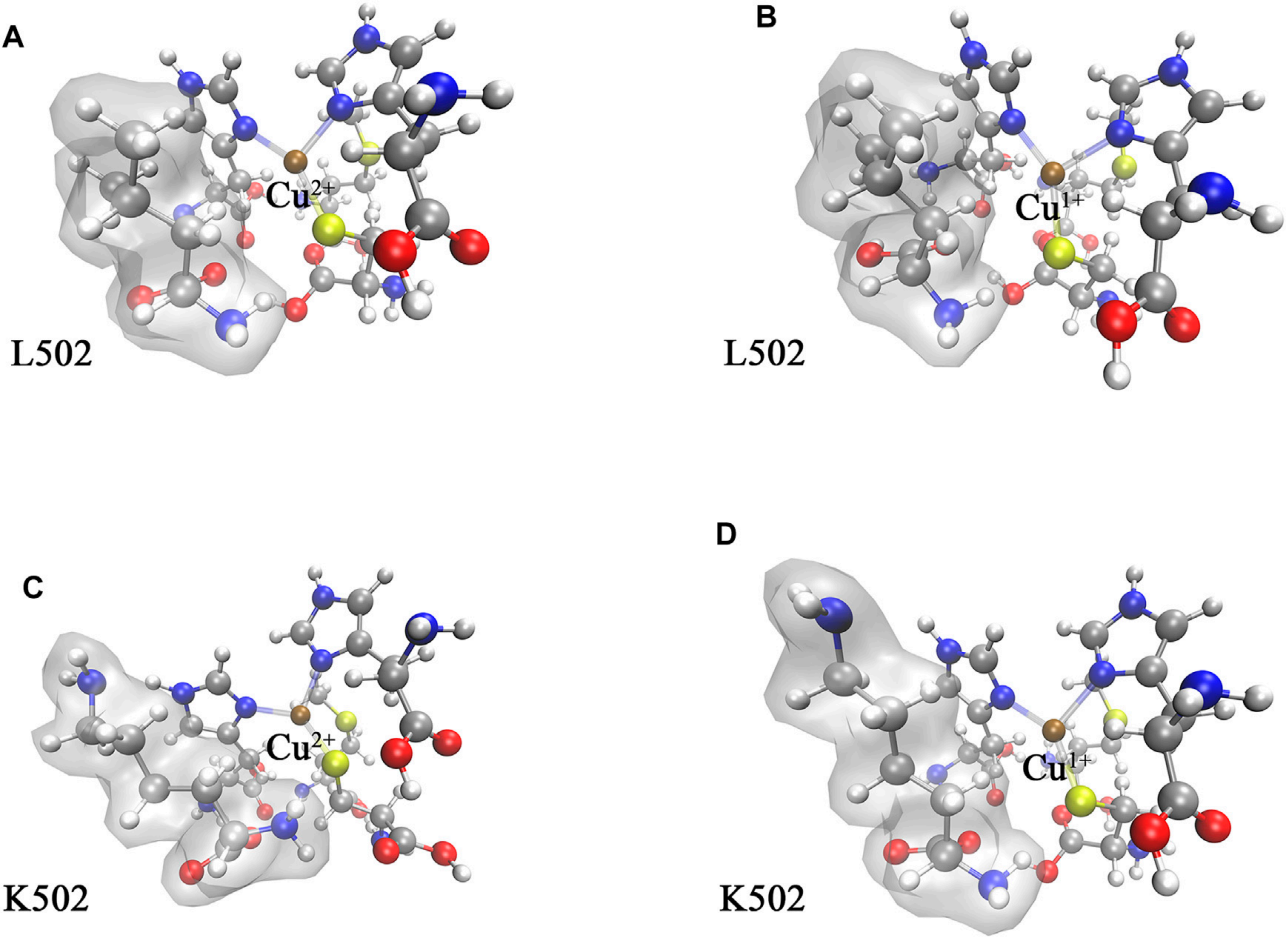
The optimized structure of CueO T1 Cu active site cluster models in the water phase: **(A)** WT in the oxidation state, **(B)** WT in the reduction state, **(C)** L502K variant in the oxidation state, and **(D)** L502K in the reduction state. The colors correspond to atoms: brown for copper, yellow for sulfur, red for oxygen, blue for nitrogen, dark gray for carbon, and light gray for hydrogen. The amino acid residue 502 was highlighted by adding a transparent molecular surface.

The optimized structures of the WT CueO and its variant cluster models are displayed in Figure 3. Owing to the conserved cupredoxin-like domain, the changes of bond distances between copper ion and coordination atom were small (less than 0.1 Å), and it was speculated that the changes of electron density around the T1 Cu site stemmed from capturing an electron majorly affected redox potentials.

Table 1 summarizes the crucial energies involved calculating the redox potential. The gas phase Gibbs free energy changes of the redox processes were calculated using the Shermo software (Lu and Chen, 2021). The gas phase free energy difference of WT and variant is −132.35 and −118.69 kcal/mol, respectively. In solution, according to the electric field intensity near the copper complexes, the copper complexes were solvated, thus exhibiting different solvation free energies. The final results showed that the Gibbs free energy variation of variant gaining one electronic is 22.92 kcal/mol higher than WT.

**TABLE 1:** DFT-calculated energetics for the WT CueO and L502K active sites and for the redox reactions (the unit is kcal/mol).

	WT	Variant (L502K)
*G*_*g*_(*ox*)	−2949799.37	−2984537.94
*G*_*g*_(*red*)	−2949931.72	−2984656.64
*ΔG*_*g*_	−132.35	−118.69
*ΔG*_*sol*_(*ox*)	−122.04	−88.45
*ΔG*_*sol*_(*red*)	−82.05	−85.04
*ΔΔG*_*sol*_	40.00	3.42
*ΔG*	−92.35	−115.27

### Redox Potentials of the Active Site

To obtain theoretical values that could be compared with experimental values, the computed $E_{\mathrm{clac}}$ value is written as $E_{\mathrm{clac}}=\mathrm{\Delta E}-\Delta E_{H}$ , where $\Delta E_{H}$ is the standard hydrogen electrode potential. $\Delta E_{H}$ is commonly used as a primary reference electrode to know the relative potentials of other redox reactions. Unfortunately, there is no universal agreement on the assigned $\Delta E_{H}$ value, which proposed the range from 4.28 to 4.74 V (Isse and Gennaro, 2010; Marenich et al., 2014). In order to avoid this uncertainty, we used the value 4.44 V for water, provided by the IUPAC24. The theoretical values $E_{\mathrm{clac}}$ at the active sites relative versus $\Delta E_{H}$ accessed by subtracting 4.44 eV from ΔE are summarized in Table 2, as well as the experimental values obtained by Zhang et al. (2019).

**TABLE 2:** Redox potentials of the active site (the unit is V).

	WT	Variant (L502K)
*E*_*exp*_	0.35	0.44
*ΔE*	4.00	5.00
*E*_*calc*_	−0.44	0.56

When compared to earlier results, this DFT research estimated redox potentials qualitatively match with the experiment. It has previously been presented that the redox potential values of copper-containing oxidases depend on the T1 Cu pocket (Kojima et al., 1990). Depending on the structure of the native high potential laccase (*T. versicolor*), Klaus Piontek et al. (2002) proposed a mechanism that assumes a decrease in electron density contribution at the metal cation through a stretching of the bond between the metal and the ligating amino acid. This mechanism could possibly explain why redox potential increased after the L502K variant. Furthermore, variant-induced structural perturbations on the electron transfer pathway were interpreted as an additional structural determinant (Xu et al., 1998).

## Conclusion

In the present work, we investigated the feasibility of cluster models to calculate the redox potential of WT laccase CueO and its variant L502K based on the Gibbs free energy. The redox potential of WT CueO was 0.79 V lower than the experimental values, while the redox potential of L502K was 0.12 V higher than the experimental values. Although the cluster method is not sufficiently accurate for determining absolute redox potentials, the trend of variant-induced redox potential changes is consistent with experimental data. Gibbs free energy variation is a function of state; from the perspective of the structures, the structural changes caused by the variant eventually led to a change in the Gibbs free energy, which led to changes in the redox potential.

Due to the atomic range limitation of the quantitative calculation, the variant sites slightly far from the active site cannot be predicted using the cluster model. With the development of computational methods, the cluster model or the QM/MM method will be able to investigate a broader range of simulations in the future, yielding more accurate results.

## Data Availability

The datasets presented in this study can be found in online repositories. The names of the repository/repositories and accession number(s) can be found below: http://www.wwpdb.org/, 3OD3.
